# Bone Quality-Independent Tibial Fixation Technique in Anterior Cruciate Ligament Reconstruction

**DOI:** 10.1016/j.eats.2025.103619

**Published:** 2025-05-16

**Authors:** Nirav H. Amin, Eric H. Lin, Cailan L. Feingold, Alexander P. Decilveo, David Flanigan, Joseph N. Liu

**Affiliations:** a Premier Orthopaedic and Trauma Specialists, Pomona, California, U.S.A.; b Keck School of Medicine of the University of Southern California, Department of Orthopaedic Surgery, Los Angeles, California, U.S.A.; c Department of Orthopaedic Surgery, Ohio State University, Columbus, Ohio, U.S.A.

## Abstract

Optimal tibial fixation in anterior cruciate ligament reconstruction is critical. Both interference and suspensory graft fixation techniques exist, each with advantages and disadvantages. This Technical Note describes a tibial fixation technique using a screw, washer, and cap system that allows complete entrapment of a soft tissue graft inside the device, allowing for interference fixation without dependance on the quality of the patient's cancellous bone.

Graft fixation, particularly tibial-sided fixation, remains a critical component to the success of anterior cruciate ligament (ACL) reconstruction surgery.^1^ Several variables contribute to tibial graft fixation, including graft tension, knee-flexion angle, and implant type.^2^ There is no clear consensus on the best implant type, with implant selection largely being patient-specific or surgeon preference.

In an ideal situation, the best graft fixation would minimize elongation, including both longitudinal (“bungee cord”) and transverse (“windshield-wiper”) type movement by maximizing strength, stiffness, stability, and durability. Multiple fixation types have been designed, including interference screws, cross pins, staples, washers, and adjustable button loops; however, the optimal fixation remains up for discussion, as it can be highly dependent on the patient's bone mineral density.

The purpose of this Technical Note is to describe a tibial ACL graft fixation technique that utilizes a screw, washer, and cap system that allows complete entrapment of a soft tissue graft inside the device, allowing for interference fixation without dependance on the quality of the patient's cancellous bone.

## Surgical Technique

### Patient Positioning

The procedure is performed under general anesthesia. The patient is placed supine on the operative table with a lateral hip post and sandbag secured with tape for positioning of the knee at 90° of flexion. Examination under anesthesia is performed, including pivot shift, Lachman, anterior/posterior drawer, and varus/valgus stress tests at 0° and 30° of knee flexion. The limb is prepped and draped in the usual sterile fashion, followed by inflation of a thigh tourniquet to 250 mm Hg.

### Graft Choice and Preparation

For this technique, any soft tissue autograft or allograft may be used. For the technique presented in this article, a double-strand semitendinosus allograft is prepared. The graft is prepped with an adjustable loop button for the femoral limb and whipstitched with high-tensile nonabsorbable suture for the 2 tibial limbs. Typical graft size is approximately 100 mm in length and 8 to 10 mm in thickness. The construct is whipstitched at approximately 15 to 20 mm from the graft edges. The graft is then measured to determine the diameter of femoral and tibial sockets.

Standard anterolateral and anteromedial arthroscopic portals are made using a No. 11 blade. A diagnostic arthroscopy is performed to identify any associated cartilage or meniscal injuries. The femoral footprint is identified and prepped using arthroscopic cautery with preservation of the ACL remnant. The tibial ACL stump is debrided using an arthroscopic shaver and cautery.

### Femoral and Tibial Tunnel Placement

The femoral tunnel is drilled according to anatomic landmarks via the anteromedial portal at 110° of knee flexion with a flexible guide pin and flexible reamer. With the arthroscope in the anterolateral portal, a flexible beath pin is inserted into the center of ACL footprint. A 4.5-mm cannulated reamer is then inserted over the beath pin to drill the path for the femoral button to pass through. Subsequently, a low-profile flexible reamer of similar size to the graft is then used to drill the femoral tunnel. The femoral socket is typically drilled 20 to 30 mm in length. Bone debris is removed via arthroscopic shaver and suction. The beath pin is then pulled retrograde through the femoral tunnel to shuttle a passing suture. The looped end of the suture is then clamped outside of the anteromedial portal.

Next, a tibial ACL aiming guide is placed through the anteromedial portal and fixed at a 50° to 60° angle over the center of the tibial insertion of the ACL. A longitudinal incision is made using a No. 15 blade along the anteromedial aspect of the proximal tibia to allow for guidewire passage and tibial tunnel reaming. A guide pin is drilled through the tibial aiming guide. Tunnel reaming of a size similar to the graft is performed over the guide pin until the tibial plateau cortex is bypassed. Finally, the orifice of the anteromedial tibial tunnel entrance is debrided using a rongeur to ensure smooth graft passage.

### Tibial Graft Fixation

**Fig 1:**
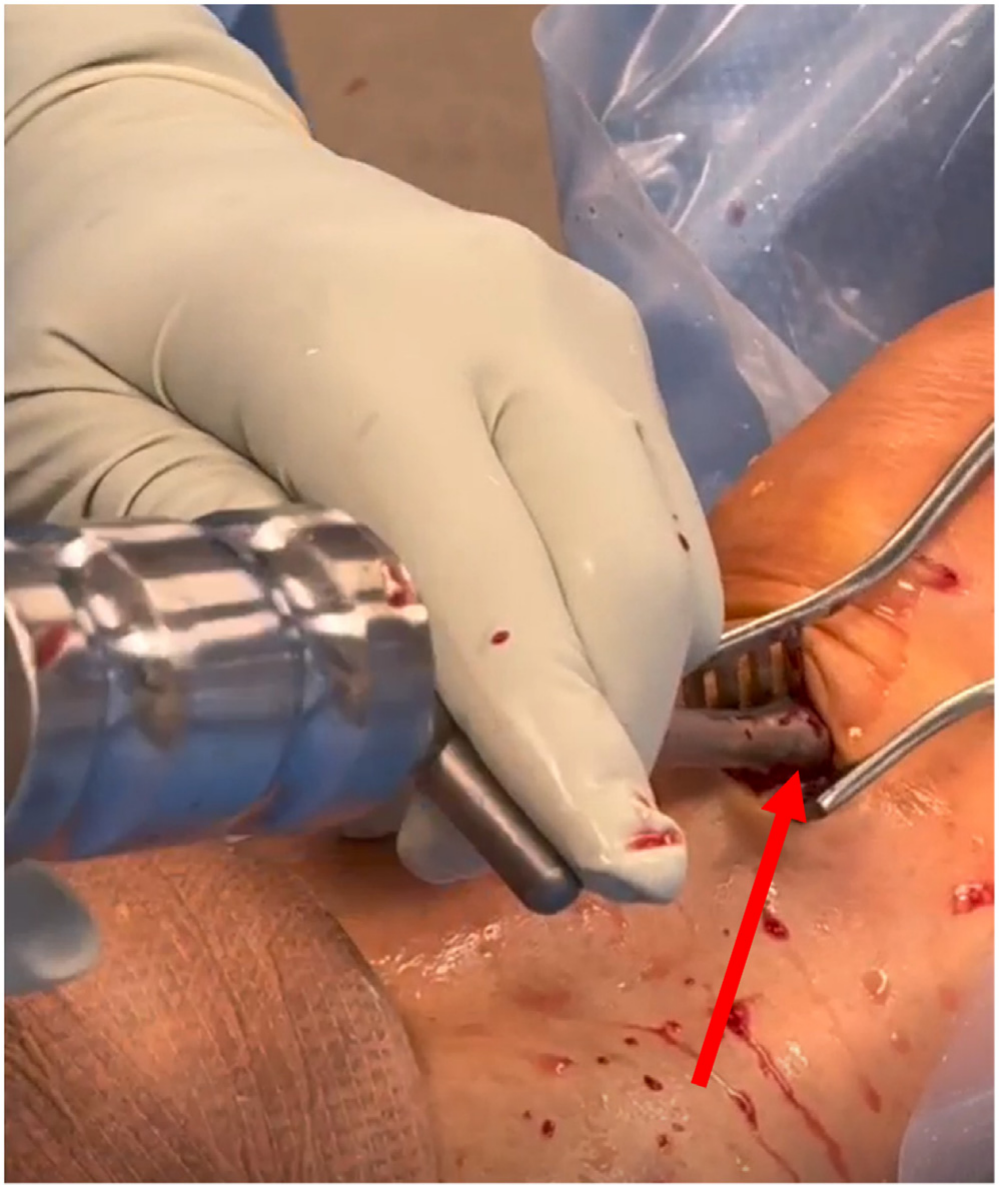
The WasherCap ACL Fixation System (Abanza) positioner is placed over the tibial tunnel and malleted into place until the lateral stoppers abut the near cortex of the tibia. The red arrow points to the end of the positioner as it is being malleted into the tibial cortex. This figure is of the right knee with the patient supine and the tibia on the left of the figure and femur and patella to the right.

Once the tibial tunnel is prepared, the WasherCap ACL Fixation System (Abanza; Mutilva Alta) positioner is placed over the tibial tunnel and malleted into place until the lateral stoppers abut the near cortex of the tibia (Fig 1, Video 1).

**Fig 2:**
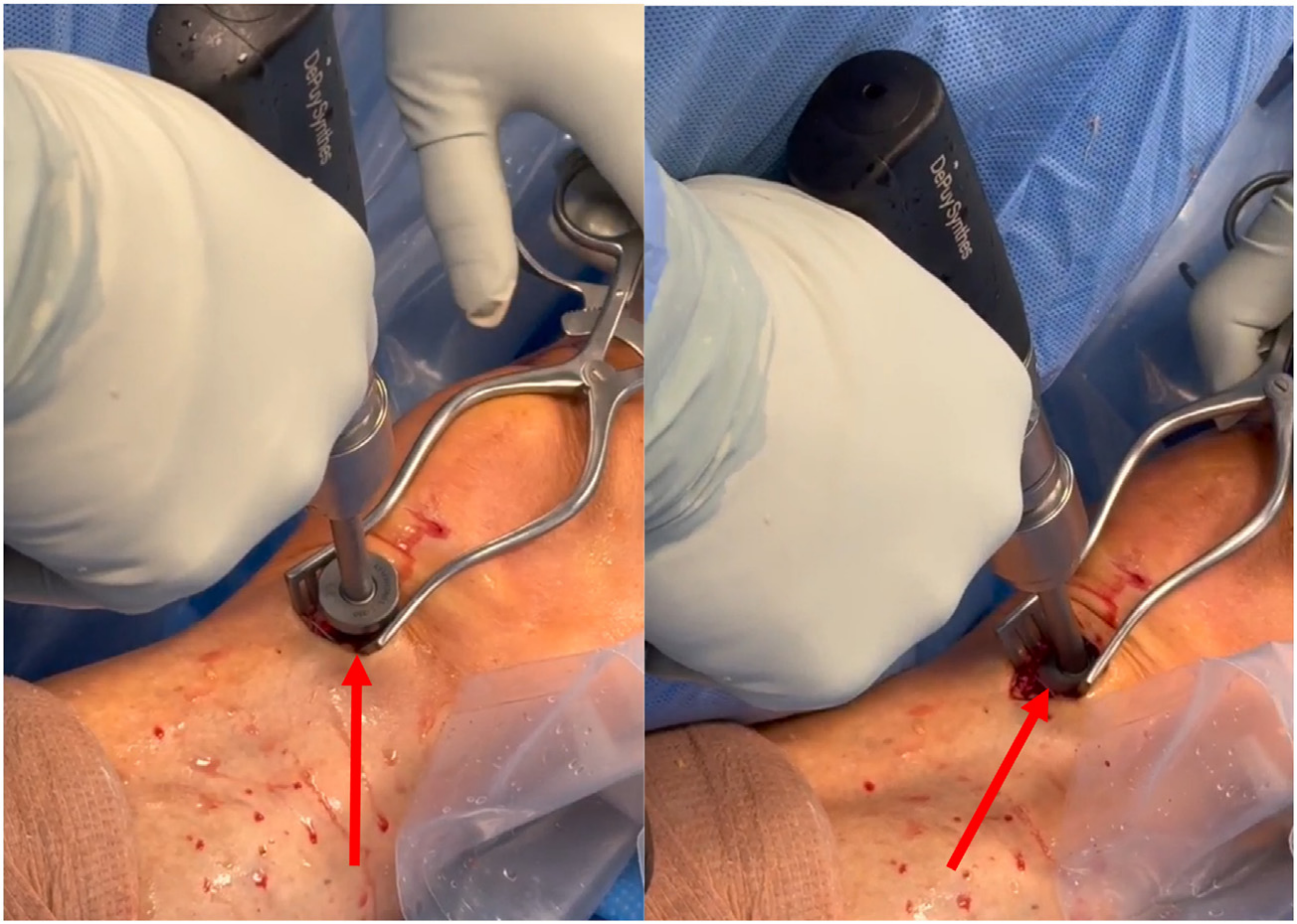
A countersink is then advanced over the wire to shape the cortex for future placement of the implant. The red arrows point to the countersink drill as it shapes the tibial cortex. These figures are of the right knee with the patient supine and the tibia on the left of each figure and femur and patella to the right.

The WasherCap guide is placed onto the positioner, and the Kirschner wire is advanced through the guide until the laser-etched line (Video 1). The guide and the positioner are then removed, leaving the Kirschner wire in place. A countersink is then advanced over the wire to shape the cortex for future placement of the implant (Fig 2).

**Fig 3:**
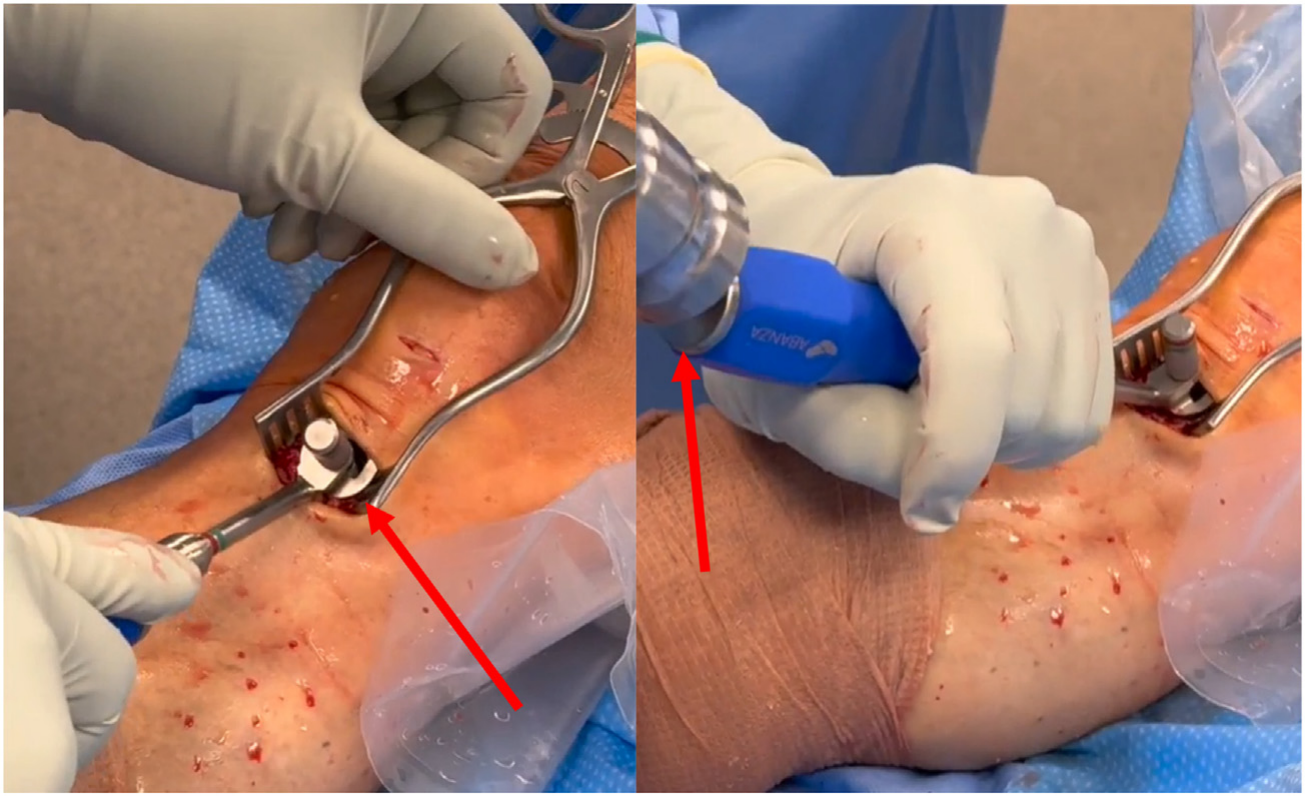
The cap is placed into the entry of the tibial tunnel and malleted until flush with the cortex. The red arrow on the left picture is showing the cap, and the red arrow on the right is pointing to the mallet. These figures are of the right knee with the patient supine and the tibia on the left of each image and femur and patella to the right.

**Fig 4:**
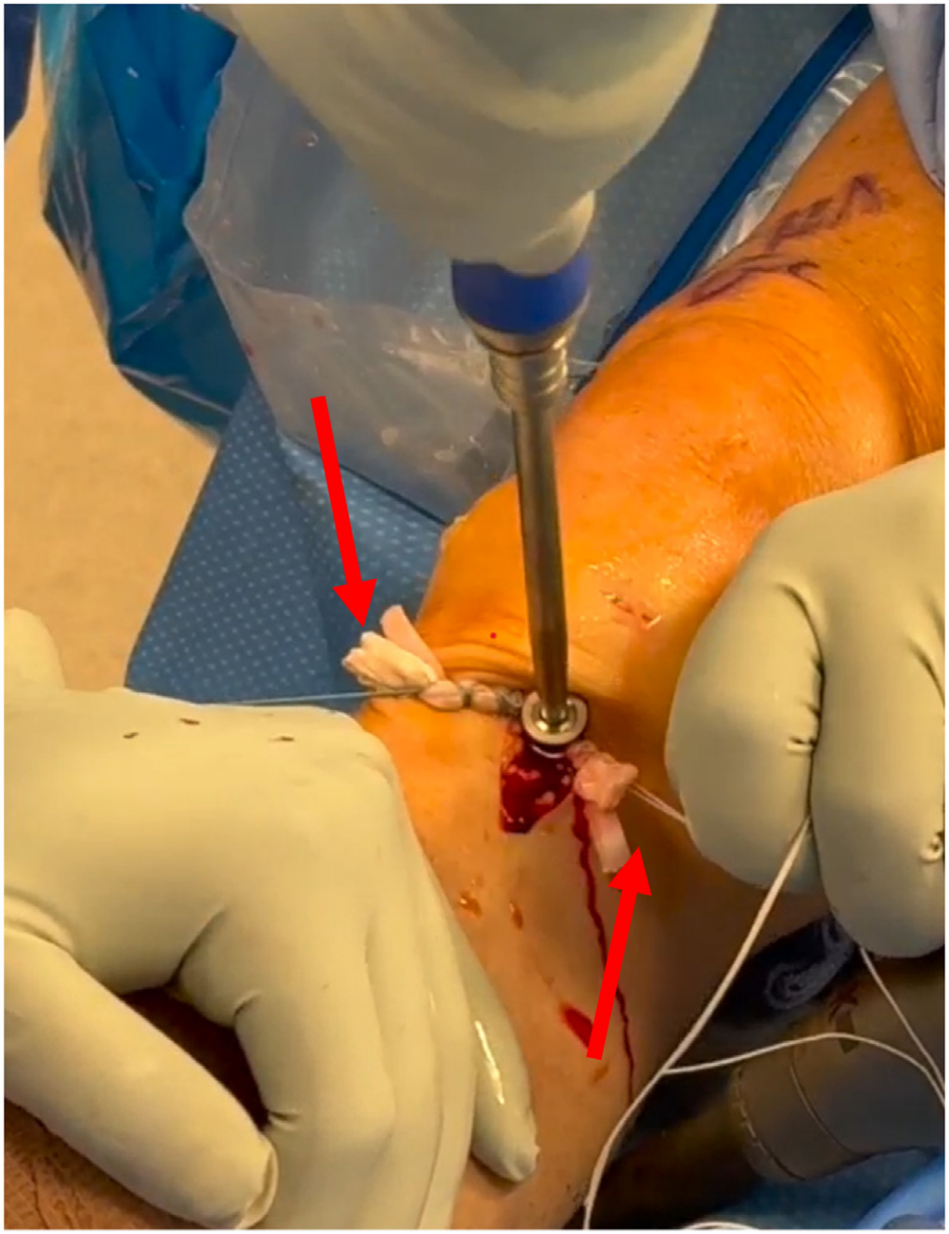
The tibial graft limbs are tensioned at the 4- and 8-o'clock positions with a simultaneous posterior drawer maneuver, and the screw and washer are advanced into the threaded hole of the cap. The red arrows point to the ends of the graft that have been cut and are being tensioned. This figure is of the right knee with the patient supine and the tibia on the left of the figure and femur and patella to the right.

Subsequently, the countersink and wire are removed. The cap is then placed and fixed over the WasherCap inserter with the coupling screw. The cap is placed into the entry of the tibial tunnel and malleted until flush with the cortex (Fig 3). The coupling screw is loosened, followed by removal of the inserter from the cap. The shuttling suture from the anteromedial portal is then retrieved through the tibial tunnel and cap with an arthroscopic grasper to allow for graft passage (Video 1). The femoral limb adjustable loop button and graft are advanced through the cap and tibial tunnel until the button is positioned against the femoral cortical surface. With inline tension on the tibial limbs of the graft, the femoral button is tensioned and secured using an arthroscopic knot tier via a small incision along the exit point of the femoral suture limbs with the knee in 90° to 110° of flexion. The knee is then placed at 20° of flexion. The tibial graft limbs are tensioned at the 4- and 8-o'clock positions with a simultaneous posterior drawer maneuver, and the screw and washer are advanced into the threaded hole of the cap (Fig 4). After the WasherCap is secured, Lachman test is performed under anesthesia to ensure adequate graft tension. This allows for adjustments to the screw and washer, if necessary; if the tension is not to the surgeons' satisfaction, the screw and washer may be removed, the graft retensioned, and the screw and washer replaced (Table 1). Excess portions of the graft are excised with a No. 15 blade once tension is satisfactory. Additional backup fixation can be used distally at surgeon discretion.

**Table 1:** Pearls and Pitfalls

Pearls	Pitfalls
• Exposing to bone can help ensure that the implant sits flush on the bone for maximum fixation. • Cyclical knee movement before fixation can remove creep. • WasherCap (Abanza) and screw can be removed and reinserted if graft is not tensioned adequately without damaging graft.	• If the graft is too bulky, tensioning the graft may dislodge the implant before final fixation. • Graft needs to be of sufficient length to allow for WasherCap to achieve fixation at the aperture of the tibial tunnel.

### Rehabilitation

Postoperatively, the patient is placed in a dynamic knee brace locked in extension. The patient can bear weight as tolerated with crutches on their operative extremity immediately. They begin physical therapy for knee range of motion with the brace unlocked. Sutures are removed at 2 weeks postoperatively, and the patient is typically advanced to a jogging/running program at 6 months postoperatively. Full return to sport is expected at 10 to 12 months postoperatively as long as there is adequate restoration of quad strength and function.

## Discussion

The WasherCap offers a stable, press-fit, tibial fixation during ACL reconstruction that is independent of bone quality. This provides an advantage in patients with poor bone quality by utilizing the WasherCap implant as the anchor point for the interference fixation.

Tibial fixation is known to be the weakest fixation of ACL reconstruction surgery, and bone density typically plays an important role in the security of this fixation.^1^ Interference screws are a popular method of graft fixation but tend to rely on bone density for graft fixation strength and carry risks of graft damage during screw insertion as well as tunnel widening.^3^ Additionally, the proximal tibia has been shown to lose bone density following ACL injury, carrying implications for reconstruction in all ACL patients, not just those with generally lower bone density.^4^

Other methods of fixation, including suspensory button systems, implant-free press-fit, and hybrid methods, have been studied as well.^5^ Suspensory devices allow for full bone-graft contact throughout, with fixation to cortical bone as opposed to cancellous bone.^6^ Implant-free fixation aims to minimize tunnel widening and graft laceration. Previous clinical studies have shown no differences in outcomes when comparing interference, suspensory, and hybrid fixation techniques.^6^^,^^7^ There are a variety of tibial fixation techniques, each with their own set of benefits and complications, and care should be taken to select the most effective technique on a patient-specific basis.

The WasherCap utilizes a cap, screw, and washer system that allows for secure graft fixation while avoiding graft threading, avoids device displacement, and provides tunnel plugging (Table 2). While the system can only be used with soft tissue grafts and requires additional steps to secure the graft compared to standard techniques, it is easily reproducible and protects the graft. This system may present a solution for tibial fixation in patients with poor bone quality.

**Table 2:** Advantages and Disadvantages

Advantages	Disadvantages
• Fixation is independent of bone quality and is especially useful in osteoporotic bone • Avoids graft threading and protects graft • Provides tunnel plugging • Easily reproducible technique	• Can only be performed with soft tissue grafts on the tibial side • Additional steps needed to secure implant compared to standard anterior cruciate ligament techniques

## Funding

D.F. has received research support from Aesculap/B.Braun, Anika Therapeutics, Arthrex, Cartiheal, CartiLife, Ceterix, Episurf, Moximed, Musculoskeletal Transplant Foundation, Smith & Nephew, Stryker, Vericel, and Zimmer.

## Supplementary Data

Video 1 Use of a bone quality–independent tibial fixation washer-cap system technique for anterior cruciate ligament reconstruction.
